# A Case of Subungual Exostosis With Early Postoperative Recurrence and Rapid Growth

**DOI:** 10.7759/cureus.73933

**Published:** 2024-11-18

**Authors:** Shohei Murata, Hiroyuki Tsuchie, Hiroyuki Nagasawa, Toshihito Ebina, Naohisa Miyakoshi

**Affiliations:** 1 Orthopaedic Surgery, Akita University Graduate School of Medicine, Akita, JPN; 2 Orthopedic Surgery, Akita University Graduate School of Medicine, Akita, JPN; 3 Orthopaedic Surgery, Akita University Graduate School Of Medecine, Akita, JPN; 4 Orthopedic Surgery, Kakunodate General Hospital, Senboku, JPN

**Keywords:** chronological radiographic progression, hallux, postoperative recurrence, subungual exostosis, surgical excision

## Abstract

Subungual exostosis is a relatively rare benign bone tumor that occurs near the distal phalanges of the fingers and toes. Though it is similar to osteochondroma, it can be distinguished by its specific location and lack of communication with the medullary cavity. Although recurrence after surgical excision has been reported, no studies have examined the timing or progression of recurrence, nor have there been reports detailing the chronological imaging findings of recurrent cases. A case of subungual exostosis that recurred rapidly with significant growth shortly after surgical excision is reported. The patient was a 13-year-old boy with subungual exostosis of the fourth toe. Tumor recurrence was observed four weeks after the initial excision, and it gradually enlarged, growing to several times the size of the original tumor three months after surgery. A second procedure was performed approximately four months after the initial procedure, and no recurrence has been observed since. This case represents the first report of the chronological radiographic progression of recurrent subungual exostosis, highlighting the importance of careful follow-up and consideration of early recurrence after excision.

## Introduction

Subungual exostosis is a relatively rare benign bone tumor that occurs near the distal phalanges of the fingers and toes [1]. It is characterized by bone trabeculae transitioning through a cartilaginous cap and enchondral ossification, resembling osteochondroma. However, it can be distinguished from osteochondroma by its specific location and lack of communication with the medullary cavity [2]. The standard treatment is surgical excision of the tumor, but recurrence is not uncommon, and there are reports of recurrence [3]. However, no studies have thoroughly investigated the timing of recurrence, nor have there been reports of the sequential imaging findings of recurrent cases. A case of subungual exostosis that recurred rapidly with significant growth shortly after surgical excision is reported.

## Case presentation

**Figure 1:**
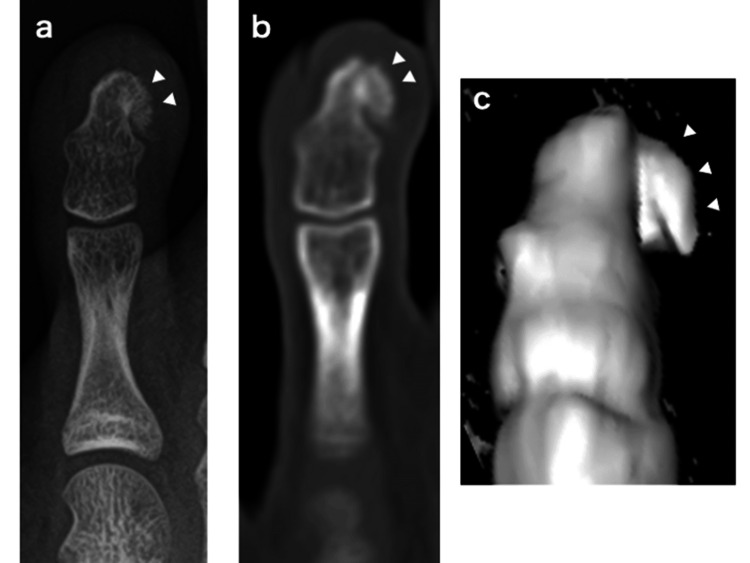
Pre-operative X-ray (a), CT (b), and 3DCT (c) images. The arrow heads indicate the tumor.

**Figure 2:**
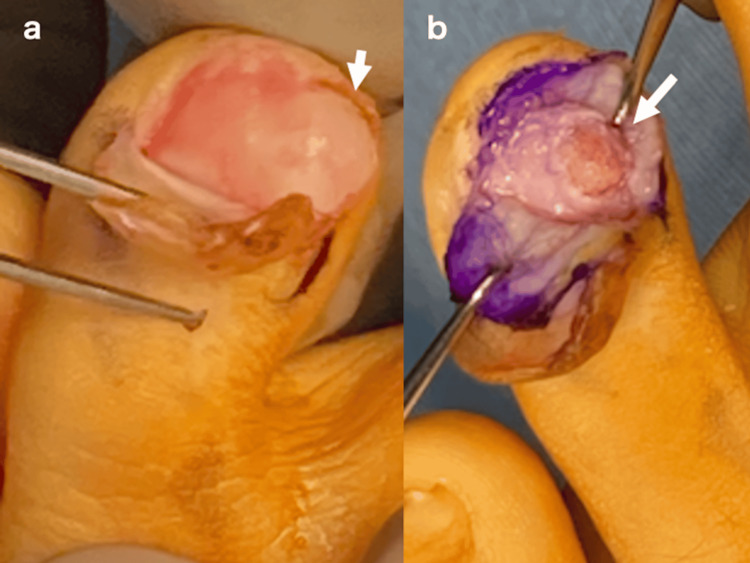
Gross photograph at initial surgery. Tumor (white arrow) perforates the nail bed (a). After excision of the tumor, the base of the tumor is curetted until the cancellous bone (white arrow) is exposed (b).

**Figure 3:**
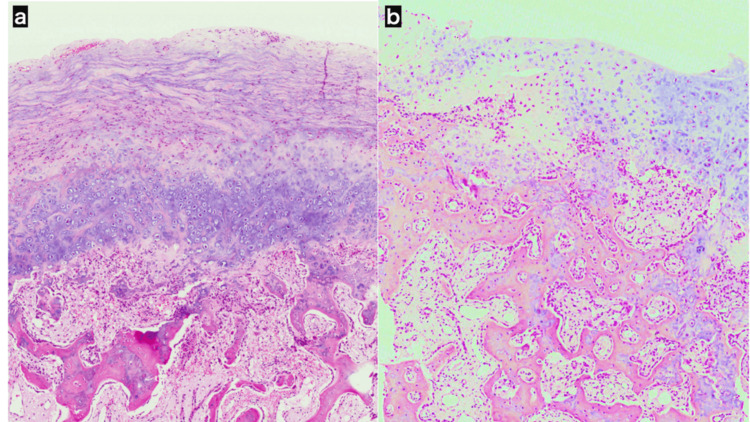
Light microscopic images of histological specimens. The histological specimen of the tumor excised during the initial surgery showed dermal tissue with normal trabecular bone and fibrocartilaginous overgrowth, consistent with subungual exostosis (a). The histological specimen from the tumor removed during the second surgery similarly demonstrated dermal tissue with overgrowth of bony trabeculae and fibrous cartilage, akin to the findings from the initial surgery, and was diagnosed as a recurrence of subungual exostosis (b).

The patient was a 13-year-old boy who presented to our hospital with swelling and pain in the left fourth toe, which he had first noticed two months prior during baseball practice. On initial examination, a bony prominence was observed beneath the nail of the distal phalanx. Computed tomography (CT) showed a bone tumor extending from the distal phalanx toward the medial side of the toe (Figure 1). Suspecting subungual exostosis, the tumor was surgically excised one month after the initial consultation. Upon elevating the nail, the cartilaginous cap of the tumor was found to have perforated and exposed the nail bed (Figure 2a). The cartilaginous cap and bony components of the tumor were resected from the base, followed by curettage until the cancellous bone was exposed (Figure 2b). A pathological diagnosis of subungual exostosis was made (Figure 3a).

**Figure 4:**
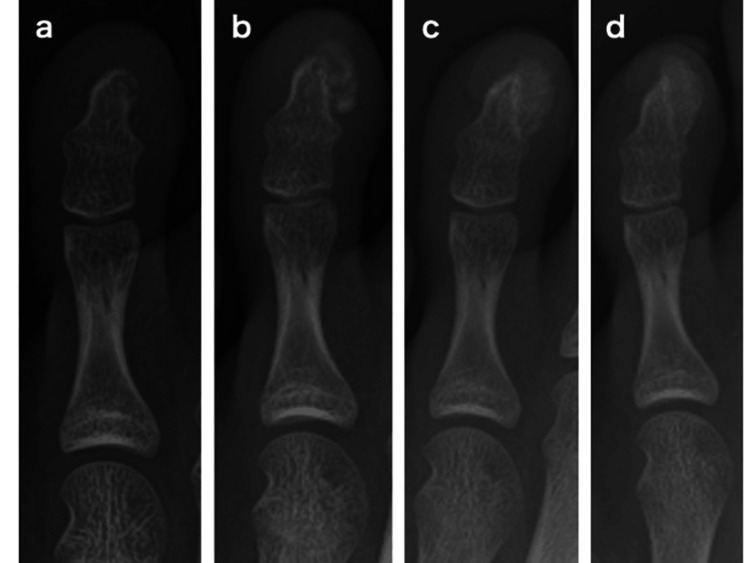
Postoperative X-rays. Postoperative X-rays, at 1 week (a), 4 weeks (b), 8 weeks (c), and 12 weeks (d). At around 4 weeks after surgery, radiographs show pale calcification in an area larger than the original bone tumor (b). Thereafter, the calcification gradually expands in the resected area, and at 3 months after surgery, the bone tumor has grown to several times the size of the original tumor (d).

**Figure 5:**
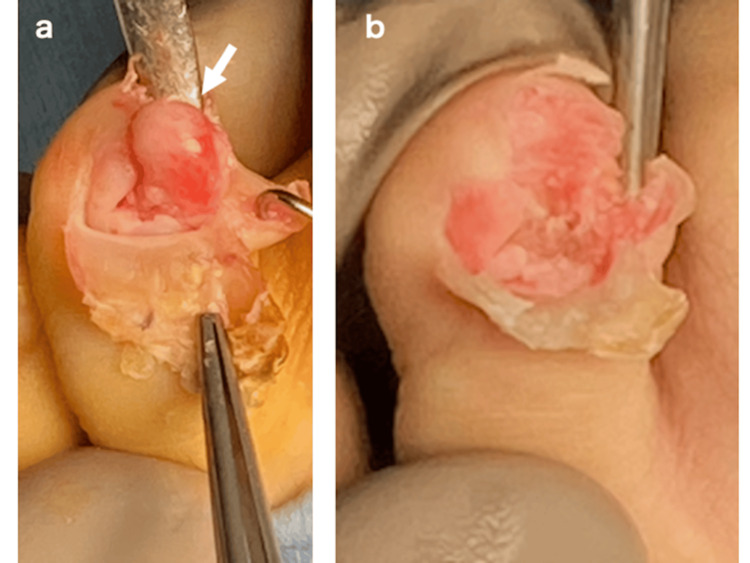
Gross photograph at the second surgery. Bony prominence with cartilaginous cap (white arrow) present (a). Thorough curettage with fluoroscopy until trabecular bone is exposed (b).

**Figure 6:**
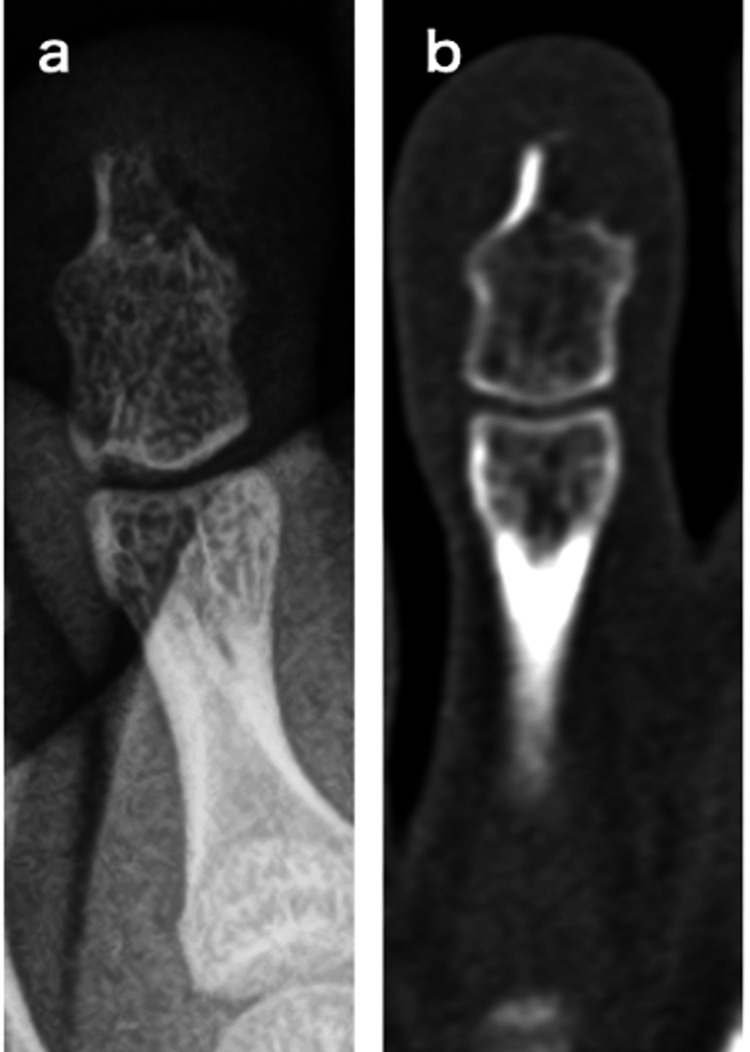
Postoperative images. Postoperative X-ray (a) and CT (b) images.

**Figure 7:**
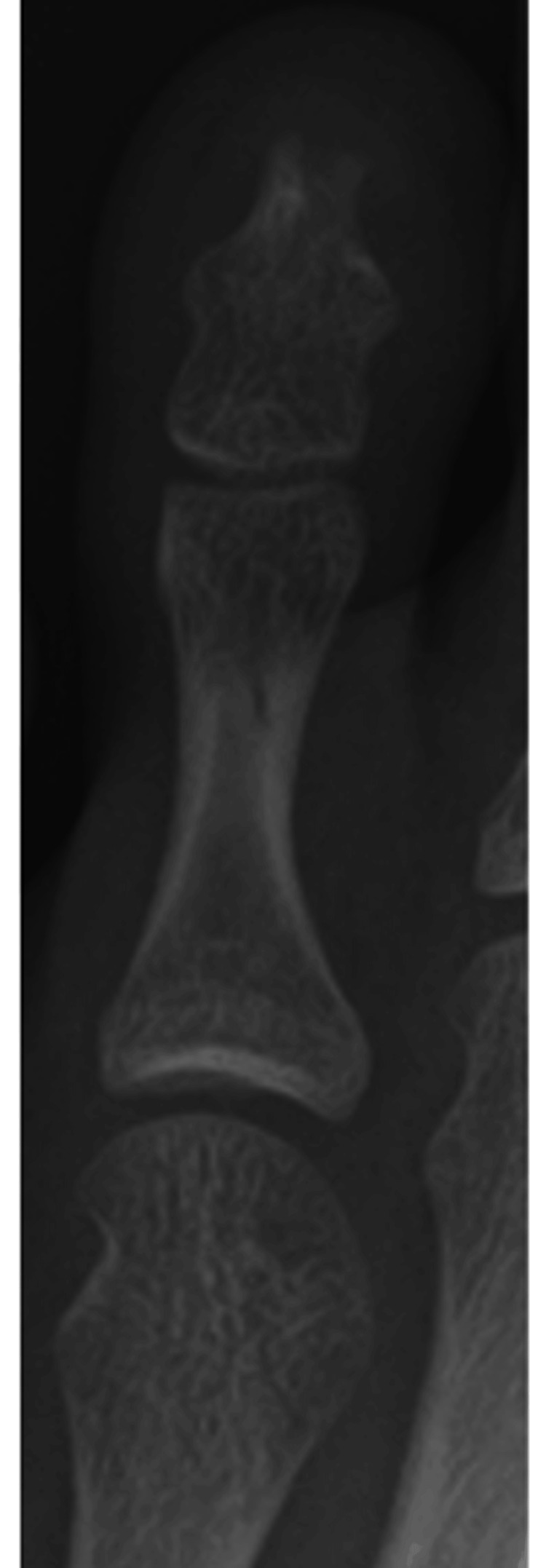
X-ray six months after revision surgery.

One week after surgery, no abnormalities were observed (Figure 4a). However, by four weeks after surgery, the patient presented with swelling at the surgical site, and plain radiographs showed faint calcification extending over a larger area than the original tumor (Figure 4b). The calcification and ossification gradually expanded over time (Figure 4c), and by three months after surgery, the tumor had grown to several times its original size and was continuous with the distal phalanx (Figure 4d). A recurrence of subungual exostosis was diagnosed, and a second excision was performed four months after the initial surgery. Upon lifting the nail, a bony tumor with a cartilaginous cap was again observed (Figure 5a). Under fluoroscopic guidance, a wide excision of the tumor, including part of the distal phalanx, was performed, followed by thorough curettage until the cancellous bone was exposed (Figures 5b, 6ab). The pathological diagnosis of subungual exostosis was confirmed at the initial surgery (Figure 3b). No recurrence was observed six months after the second surgery (Figure 7).

## Discussion

Subungual exostosis is a relatively rare condition that presents with various symptoms such as pain, onycholysis, and swelling [4,5]. The standard treatment for symptomatic subungual exostosis, such as in cases with pain, is surgical excision of the tumor [3,6,7]. The recurrence rate after surgery is reported to be around 4% [4]. It is reported that sufficient resection and curettage of the tumor to the base of the lesion is important to prevent recurrence [3,4,8]. In the present case, curettage was performed until the cancellous bone was exposed during the initial surgery, but recurrence still occurred. Although there is a report of spontaneous regression after recurrence of subungual exostosis [9], to the best of our knowledge, this is the first report documenting the sequential changes on radiographic images from recurrence to second surgery. In the present case, calcification appeared on X-rays approximately four weeks after the initial surgery, and the calcification continued to ossify until 12 weeks after surgery. At that point, it became continuous with the distal phalanx and was more significant than the original tumor. This suggests that the possibility of early recurrence and rapid tumor growth should be considered after surgical excision of subungual exostosis.

The exact cause of subungual exostosis remains unclear, but trauma, infection, tumors, and genetic abnormalities have been suggested as possible contributing factors [4,8,10,11]. It is thought that repetitive microtrauma may play a role in the pathogenesis of this condition. In the present case, the patient had a history of physical activity, and repetitive microtrauma may have contributed to the development of subungual exostosis. In addition to incomplete excision of the tumor, younger age at diagnosis has also been reported as a risk factor for postoperative recurrence [12]. The increased risk of recurrence in pediatric cases, particularly those under 18 years of age, may be attributed to factors such as rapid bone growth, the unclear boundary between normal bone and the tumor, and the relatively small size of the bone that is subject to excision [12]. In the present case, recurrence was radiographically evident four weeks after surgery, despite the fact that the patient had not engaged in vigorous physical activity or developed infection at the surgical site during this period. Therefore, the recurrence in the present case was likely due to the patient’s young age and incomplete excision and curettage during the initial surgery. During the second surgery, fluoroscopy was used to confirm the entire distal phalanx, ensuring a more thorough excision and curettage of the tumor, with no recurrence observed since.

## Conclusions

This is the first report to demonstrate the radiographic progression of tumor growth after a recurrence of subungual exostosis. When performing surgery for subungual exostosis, thorough curettage during the initial procedure and careful postoperative monitoring, with consideration of the possibility of early recurrence, are essential.

## References

[1] Landon GC, Johnson KA, Dahlin DC (1979). Subungual exostoses. J Bone Joint Surg Am.

[2] Göktay F, Atış G, Güneş P, Macit B, Çelik NS, Gürdal Kösem E (2018). Subungual exostosis and subungual osteochondromas: a description of 25 cases. Int J Dermatol.

[3] Nekkanti S, Siddartha A, Theja S (2016). A rare case of subungual exostosis of the hallux in an adolescent: a clinico-pathological review of literature. Cancer Rep Rev.

[4] DaCambra MP, Gupta SK, Ferri-de-Barros F (2014). Subungual exostosis of the toes: a systematic review. Clin Orthop Relat Res.

[5] Stamati A, Lyrtzis C, Anastasopoulos N, Paraskevas G (2024). Unveiling subungual exostosis: a case report of prolonged misdiagnosis and successful intervention. Med Rep.

[6] Malkoc M, Korkmaz O, Keskinbora M (2016). Surgical treatment of nail bed subungual exostosis. Singapore Med J.

[7] Pascoal D, Balacó I, Alves C, Cardoso PS, Ling TP, Matos G (2020). Subungual exostosis - treatment results with preservation of the nail bed. J Pediatr Orthop B.

[8] Li H, Li H, Qi X (2022). Clinical diagnosis and treatment of subungual exostosis in children. Front Pediatr.

[9] Nakamura Y, Maruyama H, Fujisawa Y (2019). Subungual exostosis with postoperative recurrence followed by spontaneous regression. J Dermatol.

[10] Weiss DT (2024). Subungual exostosis and chronic onychocryptosis: an intricate relationship - clinical review and management. Foot Ankle Surg.

[11] Tchernev G, Grigorov Y, Philipov S (2018). Subungual exostosis in a young soccer player. Open Access Maced J Med Sci.

[12] Dąbrowski M, Rusek D, Dańczak-Pazdrowska A, Litowińska A (2023). The influence of clinical factors on treatment outcome and a recurrence of surgically removed protruded subungual osteochondroma and subungual exostosis. J Clin Med.

